# The Activation and Regulation of β2 Integrins in Phagocytes and Phagocytosis

**DOI:** 10.3389/fimmu.2021.633639

**Published:** 2021-03-31

**Authors:** Hao Sun, Kangkang Zhi, Liang Hu, Zhichao Fan

**Affiliations:** ^1^ Department of Medicine, University of California, San Diego, La Jolla, CA, United States; ^2^ Department of Vascular Surgery, Changzheng Hospital, Shanghai, China; ^3^ Department of Cardiology, Cardiovascular Institute of Zhengzhou University, The First Affiliated Hospital of Zhengzhou University, Zhengzhou, China; ^4^ Department of Immunology, School of Medicine, UConn Health, Farmington, CT, United States

**Keywords:** β2 integrins, integrin activation, integrin adaptors, phagocytes, phagocytosis

## Abstract

Phagocytes, which include neutrophils, monocytes, macrophages, and dendritic cells, protect the body by removing foreign particles, bacteria, and dead or dying cells. Phagocytic integrins are greatly involved in the recognition of and adhesion to specific antigens on cells and pathogens during phagocytosis as well as the recruitment of immune cells. β2 integrins, including αLβ2, αMβ2, αXβ2, and αDβ2, are the major integrins presented on the phagocyte surface. The activation of β2 integrins is essential to the recruitment and phagocytic function of these phagocytes and is critical for the regulation of inflammation and immune defense. However, aberrant activation of β2 integrins aggravates auto-immune diseases, such as psoriasis, arthritis, and multiple sclerosis, and facilitates tumor metastasis, making them double-edged swords as candidates for therapeutic intervention. Therefore, precise regulation of phagocyte activities by targeting β2 integrins should promote their host defense functions with minimal side effects on other cells. Here, we reviewed advances in the regulatory mechanisms underlying β2 integrin inside-out signaling, as well as the roles of β2 integrin activation in phagocyte functions.

## Introduction

Phagocytosis is the mechanism by which microorganisms are engulfed and killed, and it is the main process by which immune cells disassemble pathogens to present antigens. This is important for the innate immune response and initiating adaptive immune responses. Phagocytosis is a special form of cell endocytosis, whereby cells ingest solid particles through vesicles, including microbial pathogens (1–3). While most cells are capable of phagocytosis, the professional phagocytes of the immune system, such as macrophages, monocytes, neutrophils, and dendritic cells, excel in this process (4). During phagocytic uptake, phagocytes use receptors to interact with particles and mediate signals that encapsulate the particle within the membrane, leading to complete engulfment (5, 6). Particle recognition and uptake are conducted by a receptor ligation zipper-like process that involves several types of receptors, such as integrins, Fcγ receptors (FcγRs), and scavenger receptors (1, 7).

Integrins are essential cell-surface adhesion molecules that are widely expressed on cell membranes. As cell adhesion receptors, integrins transduce intracellular and bidirectional intercellular signals (8, 9), and are crucial for immune system functions (10, 11). In recent years, great progress has been made in elucidating integrin signal transduction mechanisms in phagocytes. β2 integrins, such as complement receptor 3 (CR3, also known as integrin αMβ2, CD11b/CD18, macrophage-1 antigen, or Mac-1) and complement receptor 4 (CR4, also known as integrin αXβ2, CD11c/CD18, or p150/95), are highly expressed in phagocytes and are important for phagocytosis. This review focuses on the role of β2 integrin activation and signaling during both adhesion and phagocytosis. We highlight the inside-out signaling basis of β2 integrin function during adhesion and phagocytosis and propose that β2 integrin-mediated phagocytosis is a great model to understand functional regulation of integrins.

## β2 Integrins Expressed by Phagocytes

β2 integrins play a major role in regulating phagocyte adhesion and migration to inflamed organs and other immunological processes, such as phagocytosis (12, 13) (**Table 1**). In mammals, professional phagocytes express complement receptors, some of which are β2 integrins, such as CR3 and CR4, which are critical for anti-pathogen defense and inflammation regulation. Phagocytes like monocytes and macrophages express all four β2 integrin family members: CR3, CR4, αLβ2 (also known as CD11a/CD18, lymphocyte function-associated antigen 1, or LFA-1), and αDβ2 (CD11d/CD18) (23). The activation of β2 integrins is involved in multiple functions of phagocytes, such as cell adhesion, locomotion, exocytosis, and phagocytosis (14, 24–26). The central role of β2 integrins in immunity is highlighted by the fact that patients with leukocyte adhesion deficiency type I (LAD-I) syndrome, who lack β2 integrin expression, are particularly prone to bacterial infections (27). LAD-III (leukocyte adhesion deficiency type III) patients have mutations in kindlin-3 (an integrin binding protein) and show a deficiency in integrin β2 activation, leading to an adhesion defect of phagocytes similar to LAD-I (28). These patients end up suffering from recurrent life-threatening infections (29). Overaggressive β2 integrin activation leads to excessive inflammation and associated tissue damage (30).

**Table 1:** Distribution of β2 integrins and phenotypes of engineered gene knockout mice.

	Distribution	Phenotypes of knockout mice	
αLβ2	All leukocytes but predominates on lymphocytes	Defective adhesion and migration of neutrophils, monocytes, and macrophages; impaired neutrophil chemotaxis; a defect in TNF-α-induced neutrophil and monocyte extravasation from blood vessels; a defect in the induction of peripheral immune responses; reduced NK cytotoxicity.	(14–16)
αMβ2	Abundant on myeloid cells, monocytes/macrophages, neutrophils, NK cells, fibrocytes, mast cells, B cells, CD8+ T cells, and CD4+ γδ T cells	Defective recruitment of neutrophils and mast cells to bacterial and fungal pathogens; a defect in neutrophil binding to fibrinogen and degranulation; impaired mast cell development and innate immunity; a defect in macrophage egression from the peritoneal cavity.	(14, 15)
αXβ2	Abundant on myeloid dendritic cells, monocytes/macrophages; expressed on human NK cells and lymphocyte subpopulations	Defect in intraperitoneal recruitment and adhesive functions of monocytes and macrophages and their ability to kill/phagocytose pathogens.	(17, 18)
αDβ2	Abundant on myeloid cells, macrophages, neutrophils, and monocytes; highly expressed on human NK cells, B cells, and γδT cells	Defective macrophage retention and reduced neutrophil accumulation in the atherosclerotic lesions; defective accumulation of mononuclear cells and neutrophils in the peritoneal cavities of mice infected by *S. typhimurium*; reduced lung macrophages and increased blood neutrophils in mice with cecal ligation and puncture sepsis or LPS-induced endotoxemia.	(19–22)

Integrin αLβ2 is critical for the adhesion of blood phagocytes (such as neutrophils and monocytes) to the vascular endothelium (31–35), as well as intravascular patrolling of monocytes (36, 37) and transendothelial migration of neutrophils (38, 39). Integrin αMβ2 is involved in cell adhesion, cell migration, phagocytosis, and degranulation of phagocytes (14, 24–26, 37, 40). Integrin αMβ2 recognizes various structurally and functionally different ligands, including extracellular matrix (ECM)-associated ligands that are released from damaged cells during inflammatory responses, such as intercellular adhesion molecule 1 (ICAM-1), glycoprotein Ib-IX, and junctional adhesion molecule 3 (JAM-3) (41–45). Both αMβ2 and αXβ2 can bind to complement component iC3b and are crucial for RhoA-dependent phagocytosis in phagocytes (46–48). The differences between these two integrins have been studied in αM and αX knockout mice (**Table 1**). αMβ2 plays a major role in recruitment of polymorphonuclear neutrophil (PMN) to bacterial and fungal pathogens. αXβ2 plays a central role in monocyte- and macrophage-mediated inflammatory functions, as shown by αXβ2 deficiency that abrogated the recruitment of monocytes and macrophages to sites of inflammation or infection and reduced the ability of these cells to kill/phagocytose pathogens (17). Integrin αDβ2 is rarely expressed on peripheral blood phagocytes but is significantly up-regulated on macrophages during inflammation (e.g., atherosclerosis) (19). Integrin αDβ2 and αMβ2 show some similarities in many functions and share some ligands, such as ICAM-1, ICAM-2, ICAM-4, fibrinogen, collagen, iC3b, heparin, GPIbα, Thy-1, and plasminogen (49, 50). Recently, it was shown that β2 integrins are required for both monocyte and hematopoietic functions, and lower β2 integrin expression is associated with more severe schistosomiasis in mice (51).

β2 integrins are important for the fusion of human (52) but not mouse (53) macrophages; Macrophage fusion happens during chronic infection of persistent pathogens or encounters with nondegradable foreign objects, and results in the formation of multinucleated giant cells. Human monocyte-derived macrophage fusion was decreased ~66% upon treatment with β2 integrin-blocking antibody (52). In mouse studies, thioglycollate-elicited peritoneal macrophages from Mac-1 knockout mice showed a significant ~50% decrease in fusion compared to those from wild-type controls (53). However, thioglycollate-elicited peritoneal macrophages from wild-type mice treated with β2 integrin-blocking antibody showed a slight (~35%) but non-significant decrease of fusion compared to those without antibody treatment (53).

## Integrin Activation by Inside-Out Signaling

**Figure 1:**
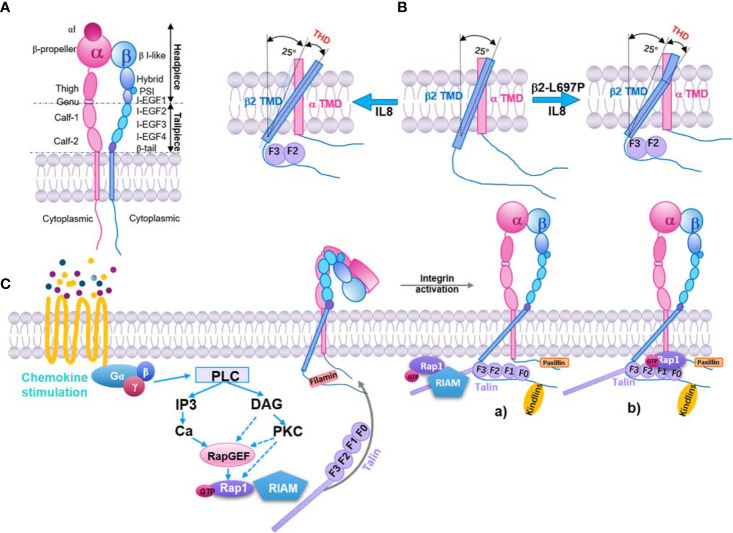
Inside-out pathway of integrin β2 activation. **(A)** Structure model of integrin β2. Subdomains and headpiece/tailpiece portions labeled. **(B)** In resting β2 integrin (middle), the beta subunit (blue) crosses the membrane at a 25° angle, whereas the α subunit (pink) crosses vertically (0 degrees). Upon exposure to IL-8 (left), talin-1 binds to the beta subunit and forces the transmembrane angle to be >25°. This change is transmitted to the extracellular domain through the stiff transmembrane domain (TMD), resulting in extended β2 integrin with an open headpiece. If the β2 TMD is mutated (β2 L697P, right), talin-1 will still bind the intracellular domain and align the beginning of the TMD to an angle >25°, but the kink prevents this from being transmitted to the extracellular domain. The integrin stays bent, but the headpiece opens (60). Talin head domain (THD). **(C)** Key signaling events that occur downstream of chemokine and lead to integrin activation. Inactive integrins exist in a bent conformation, and the α and β cytoplasmic tails are held in close proximity by a salt bridge between residues found in the membrane-proximal region of the tail. Activation of a variety of signaling pathways results in the recruitment of GTP-bound Rap1 and activated talin to the integrin, leading to tail separation. The conformational change in the cytoplasmic region is transmitted through the integrin transmembrane domains that result in structural changes in the extracellular region, leading to an open conformation that can bind ligand with high affinity. Part of this signaling pathway is shown here. **a)** The Rap1/RIAM/talin-1 axis. Rap1-GTP binds to RIAM, which leads to RIAM binding to talin-1 and recruiting of talin-1 to integrin β tails, consequently activating the integrin. **b)** The direct association of Rap1 and talin-1. Rap1-GTP binds to talin-1 through talin-F0 and F1 domains, recruiting talin-1 to interact with integrin β tails and activation of integrin.

Both integrin α and β subunits have long ectodomains with a headpiece and tailpiece, a transmembrane domain (TMD), and a flexible cytoplasmic tail (54–59) (**Figure 1A**). β2 integrins form at least three conformational states (58, 61–66): inactive (bent ectodomain with closed headpiece, bent-closed), intermediate (extended ectodomain with closed headpiece, extended-closed), and active state (extended ectodomain with open headpiece, extended-closed extended-open). The conformational change in the extracellular domains enables rapid modulation of cell adhesion and migration (58, 67, 68). The extended-open conformation in α5β1 exhibits a 4,000 to 6,000‐fold increase in ligand-binding affinity over the bent-closed and extended-closed conformations (69). On human peripheral T lymphocytes or K562 cells, most of the integrin αLβ2 are inactive. After stimulation, αLβ2 integrins on T lymphocytes are activated and show an ICAM-1 binding K_D_ of ~26 µM (~1.5-3-fold affinity increase, phorbol 12-myristate 13-acetate or stromal cell-derived factor 1 stimulation) or ~460 nM (~87-174-fold affinity increase, manganese stimulation) (65). These results indicated that only a small amount of αLβ2 integrins were activated upon leukocyte activation.

Recently, a bent-open (bent ectodomain with open headpiece) conformation was described for β2 integrins (70, 71). By introducing αX N920C and β2 V674C mutations to form a disulfide, a structure of the bent αXβ2 with an internal ligand-bound headpiece has been shown (72). The internal ligand has residues on the αI domain that can bind to the βI-like domain during activation. The binding of internal ligands is correlated to the headpiece opening in the transition from extended-closed to extended-open structure (73). The bent internal ligand-bound structure was considered a bent-open conformation of αXβ2 in this study by reviewing the structure detail of αI metal-ion-dependent adhesion site (72). There is no direct ligand-binding result of this bent internal ligand-bound integrin αXβ2. However, other mutations were introduced that are functionally relevant to the internal ligand. After Mn^2+^ treatment, the αX K313I, F315E, and I317H mutations exhibited increased monoclonal antibody 24 (mAb24) binding, which indicates headpiece opening, but unchanged KIM127 antibody binding, which indicates extension. A previous electron microscopy study showed that mAb24 exclusively binds to extended but not bent αXβ2 integrins (61). This can be explained by the different methods of expressing αXβ2 integrin protein in these two studies: Chen et al. fused αX (1-1084) and β2 (1-677) ectodomains, respectively, to a C-terminal 54-residue pepetide, which contains an acidic coiled-coil region and a cysteine for disulfide bond formation; Sen et al. introduced a disulfide bond by αX N920C and β2 V674C mutations. The difference in disulfide bond position might result in these different conformations. Thus, knowing whether bent-open β2 integrins exist on physiologically relevant cells is important.

The mAb24 and KIM127 antibodies combined with total internal reflection fluorescence microscopy or super-resolution stochastic optical reconstruction microscopy indicates the existence of the bent-open β2 integrins on primary human neutrophils (70, 71). It has been shown that β2 integrins with this conformation can bind ligands (ICAM-1, ICAM-2, ICAM-3, or Fcγ receptor IIA) expressed on the same neutrophils in *cis* and auto-inhibit neutrophil adhesion and aggregation (70, 71, 74). The cis interaction between FcγRIIA and the αI domain of bent αMβ2 (74) reduces the binding of FcγRIIA to IgG and inhibits FcγRIIA-mediated neutrophil recruitment under flow, which indicates a new anti-inflammatory function for sialylation in immune responses and benefits for auto-immune disease. Thus, cis interactions may more broadly serve as an important regulatory mechanism for calibrating both the activity of the integrin and, in turn, the heterologous receptor(s) with which it interacts. However, details of this activation mechanism need further investigation.

Intracellular proteins bind to integrin α or β subunits, lead to the separation of integrin cytoplasmic tails, and stabilize the extended-open conformation (50, 75). This can be initiated by signaling from other receptors (inside-out signaling) or ligand-binding of integrins themselves (outside-in signaling) (76). One model of integrin inside-out signaling suggests that talin (a major cytoskeletal protein; see below) binds to the β subunit cytoplasmic tail and disrupts the stabilization of the inner membrane association of α and β TMDs. This alters the membrane-crossing angle of β TMD, thereby disrupting the outer membrane association of α and β TMDs, which is important for αIIbβ3 integrin activation (77). Studies showed that these transmitting conformation changes across the cell membrane are also important for both β7 (78) and β2 integrins (60). Blocking TMD topology transmission by introducing a TMD kink (L697P mutation) impairs chemokine-induced cell adhesion and β2 integrin extension, but not chemokine-induced β2 integrin high‐affinity confirmation and manganese-induced cell spreading (60). As expected, talin-1 knockout cells showed a dramatic defect in chemokine-induced β2 integrin extension and high‐affinity confirmation as well as manganese-induced cell spreading (**Figure 1B**). These results indicate that talin-1 interaction with the cytoplasmic tail of β2 subunits may be involved in two signaling pathways: one includes the TMD topology transmission and β2 integrin extension, the other is irrelevant to the TMD topology transmission and regulates β2 integrin high‐affinity confirmation.

## Adaptor Proteins/Regulators of Integrin Activation

Integrin inside-out signaling is regulated by intracellular signaling cascades initiated from several receptors (79). In phagocytes, these receptors are mostly G-protein-coupled receptors (GPCRs) for chemokines (such as interleukin 8, monocyte chemoattractant protein-1, stromal cell-derived factor 1), cytokines (such as tumor necrosis factor α), and inflammatory factors (such as N-formylmethionyl-leucyl-phenylalanine and leukotriene B4). The canonical inside-out signaling pathway of integrin activation (50) involves the dissociation of guanine nucleotide-binding protein, the activation of Rho GTPases and phospholipases, the elevation of intracellular calcium and diacylglycerol, the activation of Ras-related protein 1 guanine nucleotide exchange factors (Rap1-GEFs) or protein kinase C, and the activation of Ras-related protein 1 (Rap-1, from GDP-bound form to GTP-bound form). Rap1-GTP can bind with Rap1-GTP-interacting-adaptor molecule (RIAM, also known as Amyloid Beta Precursor Protein Binding Family B Member 1 Interacting Protein, *APBB1IP*) and recruit talin-1 to the plasma membrane to interact with the β2 cytoplasmic tail (**Figure 1C**). Kindlin-3 is also involved in this process (80).

Rap1 is a small GTPase that functions as the hub in integrin inside-out signaling (81, 82). Rap1-dependent αMβ2 activation is critical for complement-mediated phagocytosis of red blood cells (83). Rap1 continuously circulates between inactivated (GDP-bound) and activated (GTP-bound) forms. It is activated by Rap1-GEFs from the GDP-bound form to the GTP-bound form downstream of GPCR signaling, resulting in β2 integrin activation (81, 82). Calcium and diacylglycerol regulated guanine nucleotide exchange factor I (CalDAG−GEFI) (84, 85), RapGEF1, RapGEF3, and RapGEF6 (79) have been identified as Rap1-GEFs that can activate Rap-1 and integrins. Activated Rap-1 then goes through a conformational change, allowing both recruitment and binding to its effectors.

Talin-1 is an adaptor protein linking β2 integrins to the cytoskeleton. Talin-1 has a head domain and a rod domain. The talin-1 head domain (THD) is a FERM (band 4.1, ezrin, radixin, and moesin) domain with four subdomains: F0, F1, F2, and F3. Structural studies revealed that the F3 subdomain binds to the cytoplasmic tail of β2 integrins, leading to integrin conformational change, the critical final step of integrin activation (86–90). There are two F3 subdomain binding sites in the cytoplasmic tail of β2 integrins (88): the membrane-distal binding site is the membrane-proximal NPXY motif of the β2 tail, which contains two NPXY motifs; The membrane-proximal binding site might be Y713 and F716 in β2 (corresponding to F727 and F730 in β3). Talin-1 W359A and L325R mutations cause a deficiency in binding to these two sites, respectively, and affect β2 integrin activation and neutrophil adhesion (91). The rod domain has 13 subdomains (R1-R13), including a dimerization domain and binding sites for integrin, F-actin, vinculin, and RIAM (87, 92).

In the phagocytosis of red blood cells by macrophages, talin-1 is recruited to the phagocytic cups and is essential for red blood cell capturing and phagocytosis during αMβ2-dependent uptake. Mutation of the membrane-proximal NPXY motif of the β2 tail prevents the recruitment of talin-1 to phagocytic cups as well as red blood cell phagocytosis (93). The mechanism of talin-1 activation remains unclear. A study showed that phosphatidylinositol-4-phosphate 5-kinase type 1 γ (PIP5K1γ) interacts with THD *via* a short amino acid sequence present in its 28 amino acid tail (94, 95). This interaction increases the activity of PIP5K1γ (95). Phosphatidylinositol-4,5-bisphosphate (PI(4,5)P2) is the product of PIP5K1γ and strengthens the binding of talin-1 to integrins (96). Additionally, the RIAM-talin-1 interaction is considered important for the activation and integrin tail recruitment of talin-1 (97) (**Figure 1C**). In a study using the fibroblast-like COS-7 cell line, Rap1 was found co-immunoprecipitated with talin-1 and regulated the recruitment of talin-1 to phagocytic cups. Disrupting the interaction between talin-1 and the β2 tail also inhibits the recruitment of Rap1 to phagocytic cups. Thus, Rap1 and talin-1 influence each other’s recruitment to phagocytic cups (98). Recently, a direct interaction binding site of Rap1 was found in F0 and F1 subdomains of THD (99). Synergistic interaction between these two domains and an F1 lipid-interacting helix facilitates talin-1 recognition and activation of integrins (100). This pathway could be relevant to rapid immune cell responses. Blocking direct binding between Rap1 and talin-1 inhibits neutrophil adhesion and phagocytosis but not macrophage adhesion and spreading (101, 102).

The connection between the Rap proteins and talin-1 is not fully investigated. One model suggests that activated Rap1 can recruit RIAM, which relays Rap1 signaling to talin-1 and targets talin-1 to the integrin (80); RIAM is another critical intracellular protein for integrin activation. RIAM recruits talin-1 to the cytoplasmic membrane and facilitates the binding of talin-1 and the integrin β chain (80). Deletion of RIAM results in β2 integrin inactivation, which disables β2-mediated cell migration and adhesion (103). Loss of RIAM in leukocytes prevents antigen-dependent autoimmunity by disrupting cell-cell conjugation between effector T-cells and dendritic cells (104). Recent work shows that RIAM is necessary for leukocyte integrin activation in conventional T cells. Surprisingly, it is dispensable for integrin activation in regulatory T cells, which is because lamellipodin (Lpd), a RIAM paralogue (105), compensates for RIAM deficiency (106). Lpd also contains talin binding sites and can drive integrin activation in a Rap1- and talin-dependent manner (97, 107). Interestingly, RIAM was also shown to associate with kindlin-3, even before it bound to talin-1 (108). However, whether RIAM directly interacts with kindlin-3 is unknown.

The cytoplasmic tail of β2 integrins interacts with both talin-1 and kindlin-3 (109), both important for phagocyte function. As mentioned above, talin-1 is critical for β2 integrin activation, thus essential for phagocyte adhesion and trafficking (91, 110, 111). Kindlin-3 binds to the membrane-distal NPXY motif of the β2 tail and is also vital for β2 integrin activation (112), especially the headpiece-open conformation and phagocyte adhesion (111, 113, 114). The migration and phagocytosis of macrophages are regulated by the kindlin-3 association with the cytoskeleton (115). In contrast to other known kindlin binding partners, interactions between kindlin-3 and paxillin negatively regulate integrin-dependent functions of myeloid cells and limit myeloid cell motility and phagocytosis (115). However, talin-1 and kindlin-3 play distinct roles. Talin-1 is essential for both integrin extension and headpiece-open conformation, which mediates cell slow-rolling and firm adhesion. In contrast, kindlin-3 is necessary for headpiece-open activation, which mediates firm cell adhesion (90, 111, 116). However, although both talin-1 and kindlin-3 are essential for integrin inside-out signaling, it is unclear whether they bind sequentially or simultaneously. The signaling pathway guiding kindlin-3 to integrins requires further investigation.

Additionally, many other direct or indirect integrin-tail-binding proteins, such as vinculin, filamin A, paxillin, coronin 1A, or Dok1 might be important for integrin activation regulation (76, 79, 106). Filamin A is a cytoskeletal protein that occupies the same site as talin; therefore, it negatively regulates integrin activation by blocking talin-1 binding to β integrin tails (117–119). The kindlin binding protein, migfilin, binds to filamin A. It is possible that kindlin-3 binding to migfilin releases filamin A from this binding site, leaving it free for talin (119). Thus, the shuttling on and off of filamin A from integrins may have the ability of kindlins to coactivate integrins. Several other FERM domain-containing proteins block integrin activation, such as docking protein 1 (Dok1) (120) and integrin cytoplasmic domain associated protein 1 (ICAP1), which compete for talin binding, thus blocking integrin activation (121). The talin rod domain includes actin and vinculin binding sites. It binds to the actin cytoskeleton both directly and indirectly through vinculin (122). An alternative mechanism of the Rap1/RIAM/talin1 axis was reported in lymphocytes, in which WASP family verprolin homologous 2 (WAVE2) recruited vinculin to the immunological synapse, thereby recruiting talin-1 (123). Paxillin binding to the α4 cytoplasmic tail benefits cell migration but reduces cell spreading. Phosphorylation of the integrin α4 subunit releases paxillin and the GTPase ARF6 from the membrane, leading to the accumulation of active Rac at the leading edge (124). It is worth studying these integrin-binding proteins in phagocytes to identify their roles in integrin activation and particle engulfment during phagocytosis.

## Integrin Modulation During Phagocytosis

Phagocytosis is a multi-step process. Firstly, particles are recognized and adhered to the surface of phagocytes, followed by the formation of a phagocytic cup (125), internalization, and formation of an intracellular-membrane-enclosed organelle – a phagosome (126, 127). The phagocytic cup and particle internalization is dependent on the dynamic rearrangement of F-actin, which is controlled by the Rho GTPase family (46, 128), in all forms of phagocytosis (125–127). Distinct Rho GTPases regulate several types of phagocytosis. In FcγR-dependent phagocytosis, activation of Rac1, Rac2, Cdc42, and RhoG is thought to play important roles in forming local pseudopods and membrane ruffles during particle engulfment (129, 130). Dectin-1-dependent phagocytosis involves activation of Rac1 and Cdc42, but not RhoA (131). In the FcγR and dectin-1 mediated “zipper model” mechanism of internalization, the F-actin first forms a bona fide phagocytic cup, then matures to first completely surround the bound particles and eventually fuse to complete phagocytosis (132).

αMβ2 integrin (CR3)-dependent phagocytosis exhibit distinct characteristic. The activation of αMβ2 prior to challenge with particles is required for αMβ2-mediated phagocytosis. The engulfment process in αMβ2-dependent phagocytosis is initiated by surface-tethering of particles, that then induces an invagination in the phagocyte plasma membrane into which the particle sinks, drawn by F-actin cytoskeletal forces (133). Obvious membrane ruffles were shown during αMβ2-mediated phagocytosis after integrin activation (134). These membrane ruffles differ from the membrane extensions of the zipper mechanism: They extend only from one side across the bound phagocytic particle, whereas the membrane tightly surrounds the entire surface of the particle in FcR-dependent zipper phagocytosis. Different from FcR-dependent phagocytosis, αMβ2-dependent phagocytosis requires activation of RhoA, Vav, and RhoG, but not Rac1 or Cdc42 (135, 136). However, this opinion is still controversial. Recent studies have shown that the formation of protrusions during particle engulfment is triggered by αMβ2-dependent phagocytosis (134, 137). A genetic ablation study demonstrated that Rac1 and Rac2 double-knockout macrophages are defective in both FcγR and αMβ2-mediated phagocytosis (138). This suggests that these two types of phagocytosis share common elements. Moreover, small GTPase Rap1 activation, mediated by a variety of growth factor receptors or other factors, plays an important role in αMβ2 activation and phagocytic uptake (83).

As mentioned above, talin-1 and kindlin-3 bind to the integrin β cytoplasmic tail, which activates integrins (139). Talin-1 bridges integrin with the actin cytoskeleton, stabilizes integrin activation, and transmits forces (140, 141). In the phagocytosis of red blood cells by macrophages, talin-1 is recruited to the phagocytic cups by a talin-based “molecular clutch” (142) and is essential for red blood cell capturing and phagocytosis during αMβ2-dependent uptake. Mutation of the membrane-proximal NPXY motif of the β2 tail prevents the recruitment of talin-1 to phagocytic cups as well as red blood cell phagocytosis (93). A recent study reported that β2 integrins could be coupled to actin and drive phagocytosis by a mechanosensitive molecular clutch that is mediated by talin, vinculin, and Arp2/3 (143). Thus, talin and vinculin promote phagosome formation by coupling actin to αMβ2 to drive phagocytosis. Previous studies have shown talin is transiently recruited to different types of particles during phagocytosis; however, talin is essential for αMβ2-mediated but not FcγR-mediated phagocytosis (93, 98). Kindlins are another family of integrin intracellular binding proteins that mediate integrin activation by inside-out signaling. A recent study found that kindlin-3 directly interacts with paxillin and leupaxin through its F0 domain in the macrophage-like RAW 264.7 cell line; inhibition of kindlin-3 and paxillin/leupaxin interactions promoted cell motility and augmented phagocytosis (115). Another recent work reported that kindlin-3 was essential for patrolling function and cancer particle uptake of nonclassical monocytes during tumor metastasis to the lung (144).

RIAM has been shown to play an important role in complement-dependent phagocytosis (145). Suppressing RIAM expression in neutrophil-like HL-60 cells, monocyte-like THP-1 cells, or human monocyte-derived macrophages inhibits the recruitment of talin-1 to phagocytic cups, the activation of integrin αMβ2, and complement-dependent phagocytosis (145). In RIAM knockout mice, macrophages and neutrophils show deficiencies in cell adhesion, αMβ2-mediated phagocytosis, and reactive oxygen species production (103). Recently, VASP was reported to work together with RIAM as a module regulating β2 integrin-dependent phagocytosis (146). VASP (vasodilator-stimulated phosphoprotein) is the binding partner of RIAM. This study showed that RIAM-deficient HL-60 cells presented impaired particle internalization and altered integrin downstream signaling during complement-dependent phagocytosis. Similarly, VASP deficiency completely blocked phagocytosis, while VASP overexpression increased the random movement of phagocytic particles at the cell surface, with reduced internalization. These results suggest that RIAM regulates αMβ2 activation and the cytoskeleton *via* its interaction with VASP.

## Discussion

Integrins are well-established mediators of cell adhesion and migration, yet underlying mechanisms and signaling pathways continue to be revealed (147). Further investigation is required into the role of integrins in mediating multiple phagocytic process in physiological and pathological conditions and whether integrin activation signaling pathways during cell movement and trafficking are also involved in particle engulfment.

Critical gaps remain in our knowledge of phagocytic integrin signaling. Several alternative mechanisms regulate talin-1 recruitment, but their contributions and significance are obscure. The Rap1-talin-1 interaction is evolutionarily conserved and may contribute to short-term adhesions (148), whereas the Rap1-RIAM-talin-1 axis may have longer and faster recruitment of effector proteins. Phagocytosis occurs in various cell types and is mediated by many integrin types. Several phagocytosis studies have shown that integrins need adaptor proteins or co-receptors to exert full functionality. All integrins have a common characteristic of signaling *via* Rho GTPases to modulate actin cytoskeleton dynamics. During integrin-dependent uptake, signaling involves either RhoA (for αMβ2-mediated phagocytosis) or Rac1/Cdc42 activity. This suggests that the particle engulfment in integrin-dependent phagocytosis may share similar actin-regulating pathways with general Fc-receptor-dependent phagocytosis modes.

Studies on β2 integrins indicate that integrin-mediated phagocytosis is an extension capacity of integrin-mediated cell adhesion. Besides β2 integrins, other integrins may also be involved in phagocytosis, including those in non-leukocytes. Integrins bind to ECM components, such as fibrinogen (ligand of integrin αIIbβ3, αVβ3, and others), fibronectin (ligand of α5β1, α8β1, αVβ1, αVβ3, αIIbβ3, and others), vitronectin (ligand of αvβ1, αvβ3, αvβ5, αvβ6, αvβ8, and others), or collagen (ligand of integrin α1β1, α2β1, α10β1, and α11β1). However, it is not clear which integrins are involved in phagocytosis. Those integrins known to induce actin remodeling might support particle uptake but need to be further evaluated. As far as we know, integrins αVβ3 and αVβ5 are involved in apoptotic-cell (AC) uptake (149). RGD (arginine-glycine-aspartate) peptides severely inhibit AC uptake of human macrophages (150). The remodeling of collagen is essential to the progression of a number of diseases and depends on the degradation and phagocytosis process, in which the uptake of collagen fibrils is mediated by α2β1 integrin (151).

An improved understanding of phagocytosis is important since it is involved in bacterial clearance, antigen presentation, inflammation resolution, and progression of chronic inflammatory or auto-immune diseases. β2 integrins are clearly important in phagocytosis, although their general role is just emerging. Investigating the detailed molecular mechanism of integrin functions in the complex phagocytotic process is a fascinating challenge. β2 integrins are a valuable clinical target (152). However, side effects of β2 integrin-targeting drugs include immune deficiency and infections. This may be due to the important roles that β2 integrins play in regulating the function of all kinds of immune cells, and they may exert contrary functions in a cell type-specific manner. For example, β2 integrins could limit T cell activation when expressed on antigen-presenting cells (153), but be necessary for T cell activation when expressed on T cells (154); infiltration of β2 T cells prevents tumor progression in early tumor development (155), but β2 integrins increase tumor migration and angiogenesis (156). Thus, insight into how the function of β2 integrins can be inhibited in a cell type-specific manner can avoid potential mechanism-based toxicities. This might be achieved by targeting specific integrin conformations or signaling pathways, such as if only the Rap1/talin-1 interaction pathway regulates integrin activation in platelets, the Rap1/RIAM/talin-1 axis might be dominant in lymphocytes. It is worth understanding the regulatory mechanism of β2 integrin activation in phagocytes and other cell types, since this difference can be therapeutically exploited in auto-immune diseases and cancer.

## Author Contributions

HS and KZ contributed equally to this work. HS prepared figures. HS and KZ drafted the manuscript. HS, KZ, LH, and ZF edited and revised the manuscript. ZF approved the final version of the manuscript. All authors contributed to the article and approved the submitted version.

## Funding

This research was supported by funding from the National Institutes of Health, USA (NIH, R01HL145454) and a startup fund from UConn Health.

## Conflict of Interest

The authors declare that the research was conducted in the absence of any commercial or financial relationships that could be construed as a potential conflict of interest.
